# Hyper-Aerotolerant *Campylobacter coli* from Duck Sources and Its Potential Threat to Public Health: Virulence, Antimicrobial Resistance, and Genetic Relatedness

**DOI:** 10.3390/microorganisms7110579

**Published:** 2019-11-19

**Authors:** Jae-Ho Guk, Junhyung Kim, Hyokeun Song, Jinshil Kim, Jae-Uk An, Jonghyun Kim, Sangryeol Ryu, Byeonghwa Jeon, Seongbeom Cho

**Affiliations:** 1 College of Veterinary Medicine and Research Institute for Veterinary Science, Seoul National University, Seoul 08826, Korea; gukjh@snu.ac.kr (J.-H.G.); tpkimjh@snu.ac.kr (J.K.); hsongmd@snu.ac.kr (H.S.); nsunshine@snu.ac.kr (J.-U.A.); 2 Department of Food and Animal Biotechnology, Research Institute for Agriculture and Life Sciences, Center for Food and Bioconvergence, Seoul National University, Seoul 08826, Korea; jinsilk1130@naver.com (J.K.); sangryu@snu.ac.kr (S.R.); bjeon@umn.edu (B.J.); 3 Department of Agricultural Biotechnology, Seoul National University, Seoul 08826, Korea; 4 Division of Bacterial Disease Research, Center for Infectious Diseases Research, Korea National Institute of Health, Cheongju-si, Chungcheongbuk-do 28159, Korea; micro487@hanmail.net; 5 Environmental Health Sciences, School of Public Health, University of Minnesota, Minneapolis, MN 55455, USA

**Keywords:** Hyper-aerotolerance, *Campylobacter coli*, duck sources, MLST, genetic relatedness, high-level antimicrobial resistance, virulence potential

## Abstract

*Campylobacter*, a common foodborne human pathogen, is considered sensitive to oxygen. Recently, aerotolerant (AT) *Campylobacter jejuni* with the ability to survive under aerobic stress has been reported. Here, we investigated the prevalence of hyper-aerotolerant (HAT) *Campylobacter coli* from duck sources (118 carcasses and meat) and its characteristics to assess potential impacts on public health. Half of 56 *C. coli* isolates were HAT and most harbored various virulence genes including *flaA*, *cadF*, *cdtA*, *ceuB*, and *wlaN*. Moreover, 98.2% of *C. coli* isolates showed resistance to quinolones, including ciprofloxacin (CIP), and nine (16.1%) showed high-level resistance to ciprofloxacin (Minimum Inhibitory Concentration, MIC ≥ 32 μg/mL) and most of these were HAT. Based on genetic relatedness between *C. coli* from duck sources and those from human sources (PubMLST and NCBI), HAT isolates sharing the same MLST sequence types were significantly more prevalent than those not sharing the same sequence types as those from human sources. Therefore, HAT *C. coli* is prevalent in duck sources, and is most likely transmitted to humans through the food chain given its aerotolerance. This being so, it might pose a threat to public health given its virulence and antimicrobial resistance (AMR). This study will assist in improving control strategies to reduce farm-to-table HAT *C. coli* transmission to humans.

## 1. Introduction

*Campylobacter*, especially *Campylobacter coli* and *Campylobacter jejuni*, comprises a group of the most common foodborne pathogens causing gastroenteritis symptoms such as watery or bloody diarrhea, fever, abdominal cramps, and weight loss in humans worldwide [1,2,3]. In severe cases, *Campylobacter* infections are associated with extra-gastrointestinal infections, including bacteremia, reactive arthritis, Guillain-Barré syndrome, or Miller Fisher syndrome [3]. This bacterium possesses various virulence genes associated with motility, adhesion, invasion, toxin production, expression of Guillain-Barré syndrome ganglioside mimics, and secretion due to its genetic diversity [4,5,6,7]. The use of antimicrobials—especially fluoroquinolones and tetracycline—to promote the growth of food animals that might be reservoirs of *Campylobacter* has increased antimicrobial resistance (AMR) in these bacteria [1,8]. Therefore, antimicrobial resistance in *Campylobacter* is a matter of concern [9,10].

Further, *Campylobacter* is an obligate microaerophilic organism that requires low oxygen concentrations (2–10%) for optimal growth and is considered sensitive to oxygen concentrations in the ambient atmosphere of the extra-intestinal environment [11]. However, contrary to this perception, an atypical *C. jejuni* strain that can grow aerobically [12] and aerotolerant (AT)/hyper-aerotolerant (HAT) *C. jejuni* strains that survive under vigorous aerobic shaking conditions in retail chickens [13] have been reported. These *C. jejuni* strains were found to be prevalent in retail chicken meat and most were mainly classified into major multi-locus sequence typing (MLST) clonal complexes (CCs) that are implicated in human campylobacteriosis [13]. For these reasons, concerns regarding the aerotolerance of *Campylobacter* have recently increased.

Poultry represents the most common reservoir and carrier of *Campylobacter* spp. because of the body temperature of the animal, which is optimal for the growth of *Campylobacter* [8,14]. In particular, the consumption of undercooked poultry meat or poultry products that have been cross-contaminated with raw poultry can be the main cause of human *Campylobacter* infections [15]. Among poultry, ducks are commonly consumed in Asian countries, and a potential risk of human *Campylobacter* infections associated with ducks has been reported [16,17,18]. According to previous studies, *Campylobacter coli* has been more frequently isolated from ducks than *C. jejuni* [19,20,21]. However, studies on aerotolerant *C. coli* from ducks have not been conducted.

The objectives of this study were to investigate the prevalence of HAT *C. coli* in duck sources (carcasses and meat) and its characteristics in order to assess its potential impact on public health. Toward this objective, we isolated *C. coli* from duck sources and analyzed aerotolerance levels, virulence, and antimicrobial resistance. We also compared the genetic relatedness between HAT *C. coli* isolates from duck sources and *C. coli* isolates from human sources (PubMLST and NCBI). This is the first study to assess the potential threat of HAT *C. coli* from duck sources to public health.

## 2. Materials and Methods

### 2.1. Isolation and Identification of C. coli from Duck Samples

In total, 118 duck samples (carcasses and meat) were collected from a duck slaughterhouse (*n* = 40) and retail markets (*n* = 78) from January 2017 to July 2018. Forty samples from the slaughterhouse were obtained by swabbing each duck carcass with sterilized gauzes soaked with saline. The swab samples were then applied to enrichment broth including Bolton Broth (Oxoid Ltd., England, UK) and Preston Broth (Oxoid Ltd., England, UK) and cultured at 42 °C for 24 h under microaerobic conditions (6% O_2_, 7.1% CO_2_, 3.6% H_2_, and 83.3% N_2_). For the 78 samples from retail markets, duck meat was submerged in enrichment broth and cultured at 42 °C for 24 h under microaerobic conditions. The enriched broth was streaked on modified charcoal cefoperazone deoxycholate (mCCD) agar (Oxoid Ltd., England, UK) and Preston agar and incubated at 42 °C for 24 h under microaerobic conditions. Next, *Campylobacter*-like colonies were picked, and template DNA was prepared. To identify *C. coli*, polymerase chain reaction (PCR) was performed using primers designed on *Campylobacter* 16s rDNA and *ask* genes (Table S1).

### 2.2. Analysis of the Aerotolerance Levels of C. coli Isolates

Aerotolerance tests were performed using *C. coli* isolates and a previous method with modifications [13]. The isolates were cultured on MH agar for 24 h at 42 °C under microaerobic conditions and resuspended in Mueller-Hinton broth (MH broth) to a McFarland standard of 1.0 (3 × 10^8^ CFU/mL). The bacterial suspension was incubated at 42 °C under aerobic conditions with shaking at 200 rpm. The samples were diluted serially and inoculated on MH agar for CFU counting at 0, 12, and 24 h after aerobic shaking. The isolates that died under aerobic shaking within 12 h were considered oxygen-sensitive (OS) strains, while those that survived under aerobic shaking for 12−24 h were considered aerotolerant (AT), and those that survived under aerobic shaking for more than 24 h were considered HAT strains [22]. The experiments were replicated at least three times.

### 2.3. Virulence genes of C. coli Isolates

The prevalence of 10 virulence genes was investigated using PCR with several primers (Table S1). For this, *flaA* and *flhB* were selected as virulence genes responsible for motility, *cadF* and *pldA* for adhesion, *iamA* and *ceuE* for invasion, *cdtA* for cytotoxin production, *wlaN* for association with Guillain-Barré syndrome, and *hcp* and *virB11* for association with a type VI secretion system and type IV secretion system, respectively [4,23,24,25,26,27,28,29].

### 2.4. Antimicrobial Resistance of C. coli Isolates

Antimicrobial susceptibility tests were conducted to determine the minimum inhibitory concentrations (MICs) of nine antimicrobials against *C. coli* isolates, including erythromycin (ERY), chloramphenicol (CHL), ciprofloxacin (CIP), tetracycline (TET), telithromycin (TEL), gentamicin (GEN), azithromycin (AZI), streptomycin (STR), and nalidixic acid (NAL) using the broth microdilution method with the Sensititre custom plate KRCAMP (TREK Diagnostics, Cleveland, OH, USA). Resistance to all antimicrobials except STR was determined using the interpretative standard suggested by the National Antimicrobial Resistance Monitoring System (NARMS, https://www.cdc.gov/narms/antibiotics-tested.html), and resistance to STR was determined using the epidemiological cut-off (ECOFF) values of the European Committee on Antimicrobial Susceptibility Testing (EUCAST) [30]. In addition, the distribution of MICs was investigated among resistant *C. coli* isolates to identify isolates with high-level resistance to several antimicrobials including quinolones (CIP and NAL). For quinolones (CIP and NAL), isolates associated with MIC values for CIP and NAL ≥ 32 μg/mL [31] and ≥ 64 μg/mL [32] were considered high-level CIP-resistant and NAL-resistant strains, respectively. Antimicrobial resistance (AMR) patterns including multidrug resistance (MDR) patterns, which are defined as acquired non-susceptibility to at least one agent in three or more antimicrobial categories [33], were analyzed. GraphPad Prism 7.00 was used to present the distribution of MIC values according to aerotolerance levels in *C. coli* isolates from duck sources.

### 2.5. Clonal Distribution Analysis of C. coli Isolates from Duck Sources Based on MLST Genotypes

*C. coli* isolates were genotyped by MLST according to the protocol at PubMLST (https://pubmlst.org/campylobacter/). The sequences of seven housekeeping genes (*aspA, glnA, gltA, glyA, pgm, tkt,* and *uncA*) in *C. coli* isolates were submitted to PubMLST, and the MLST sequence types (STs) were determined. *C. coli* isolates were clustered by MLST STs using the BioNumerics software Version 6.6 (APPLIED MATHS) with the minimum spanning tree (MST) method and hypothetical nodes based on allelic numbers of MLST housekeeping genes.

A dendrogram based on allele numbers of seven MLST housekeeping genes (*aspA*, *glnA*, *gltA*, *glyA*, *pgm*, *tkt*, and *uncA*) was generated to present the aerotolerance levels, virulence genes, and antimicrobial resistance of *C. coli* isolates based on each MLST ST. The dendrogram was constructed using the unweighted-pair group method with arithmetic mean (UPGMA) method of the BioNumerics software.

### 2.6. Analysis of the Genetic Relatedness between C. coli Isolates from Duck and Human Sources

To analyze genetic relatedness between *C. coli* isolates from duck and human sources, the MLST data or whole genome shotgun sequences (WGSs) of *C. coli* isolates from a human source registered in PubMLST and NCBI (National Center for Biotechnology Information, U.S. https://www.ncbi.nlm.nih.gov/) were used.

MLST data for *C. coli* isolates from a human source were downloaded from PubMLST (accessed on 28 January 2019). Of 689 MLST STs from PubMLST, 39 MLST STs containing at least 10 isolates from human sources were selected as representative isolates from humans. A neighbor-joining tree was constructed based on concatenated nucleotide sequences of seven housekeeping genes for MLST STs of *C. coli* isolates from duck sources in this study and from a human source (PubMLST) with MEGA 7. The distances in the tree were computed using the maximum composite likelihood method.

WGS data of approximately 850 human *C. coli* isolates, for which the host was identified as human and assembly data were available, were downloaded from NCBI (accessed on 2 November 2019) (Table S2). The data were analyzed to determine the MLST STs. A neighbor-joining tree was generated using the MLST STs of *C. coli* isolates from duck sources in this study and from human clinical cases in NCBI in the same manner as previously mentioned.

### 2.7. Statistical Analysis

Odds ratios (ORs) with 95% confidence intervals (CIs) were calculated to compare the proportions of HAT *C. coli* between *C. coli* sharing MLST STs with isolates from a human source and *C. coli* belonging to other MLST STs. OR and CI calculations were performed using SAS software version 9.4 (SAS Institute Inc., Cary, NC, USA). Mid-P exact tests were performed to assess differences in the levels of resistance to CIP according to aerotolerance levels of *C. coli* using SAS software version 9.4 (SAS Institute Inc., Cary, NC, USA).

## 3. Results

### 3.1. Identification of Aerotolerant C. coli Isolates from Duck Sources

Sixty-one *C. coli* strains were isolated from 55 of 118 duck samples. Of the 55 samples, six harbored two different *C. coli* strains that were epidemiologically unrelated (phenotypically or genotypically) (Tables S3 and S4). Fifty-six *C. coli* isolates were used in this study, with the exception of five *C. coli* isolates that entered a viable but non-culturable state.

In the aerotolerance test, the proportions of *C. coli* determined to be OS, AT, and HAT were 10.7% (*n* = 6), 39.3% (*n* = 22), and 50% (*n* = 28), respectively (Table S3).

### 3.2. Virulence Genes in C. coli Isolates

The genes *flaA* (85.7%), *cadF* (92.9%), *ceuE* (83.9%), *cdtA* (96.4%), and *wlaN* (92.9%) were predominant, whereas other genes including *flhB* (1.8%), *pldA* (3.6%), *iamA* (8.9%), *hcp* (8.9%), and *virB11* (3.6%) were scarce (Table 1). No significant association was observed between the proportion of virulence genes and aerotolerance in *C. coli*.

### 3.3. Antimicrobial Resistance of C. coli Isolates

Most *C. coli* isolates from duck sources were resistant to CIP (98.2%), NAL (98.2%), and TET (76.8%) (Table S5). There was no significant difference between antimicrobial resistance rates and aerotolerance levels of *C. coli* isolates in this study (Figure S1). However, we identified a tendency for HAT *C. coli* to show higher resistance levels to CIP than OS *C. coli* (Mid P exact test, *p* = 0.11). The MIC values for CIP resistance varied from 4 to 64 μg/mL (Table 2), and nine isolates among the CIP-resistant *C. coli* isolates were determined to be highly resistant to CIP (MICs ≥ 32 μg/mL). Most (77.8%) of the isolates with high-level resistance to CIP were HAT. Twenty-five percent of HAT *C. coli* isolates and 9.1% of AT *C. coli* isolates were highly resistant to CIP, whereas none of the OS *C. coli* isolates were highly resistant to CIP. All NAL-resistant *C. coli* isolates had a high-level resistance (MICs ≥ 64 μg/mL) (Table 2). Analysis revealed eight AMR patterns (Table 3). Five of the eight AMR patterns in this study were MDR patterns and 14 of the 56 *C. coli* isolates (25%) were determined to be MDR.

### 3.4. Clonal Distribution Analysis of C. coli Isolates Based on MLST Genotypes

**Figure 1:**
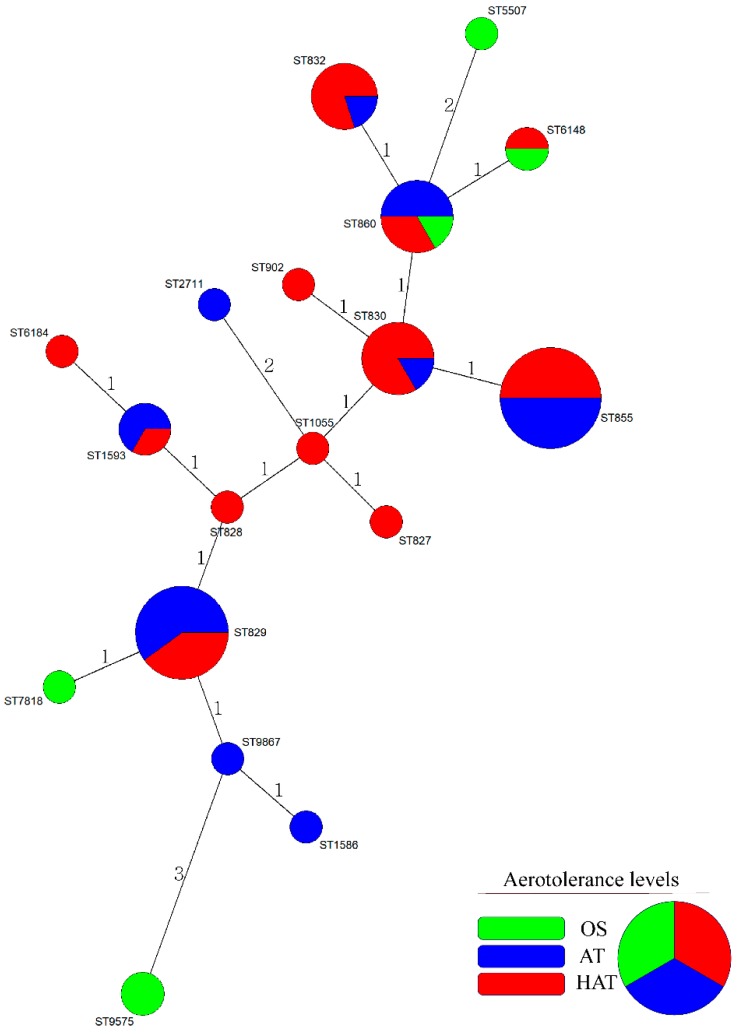
Minimum spanning tree (MST)-based cluster analysis of *C. coli* isolates according to aerotolerance levels. Cluster analysis of *C. coli* isolates from duck sources is based on multi-locus sequence typing (MLST) sequence types (STs). The analysis was performed using an MST with hypothetical nodes based on allelic numbers of seven MLST housekeeping genes. Each circle represents the MLST ST and the diameter of each circle represents strain numbers for each ST. Colors indicate aerotolerance levels of *C. coli* in each MLST ST (green, OS; blue, AT; red, HAT). OS, oxygen-sensitive; AT, aerotolerant; HAT, hyper-aerotolerant.

**Figure 2:**
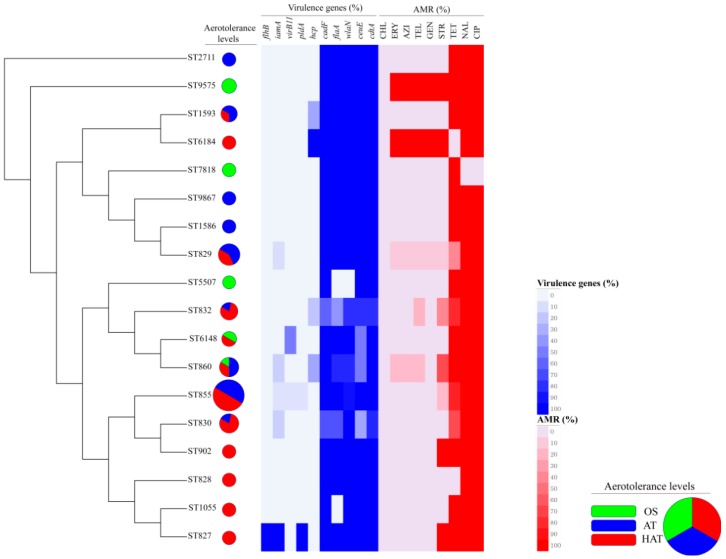
Clonal distribution of *C. coli* isolates by multi-locus sequence typing (MLST) sequence types (STs). The unweighted-pair group method with arithmetic mean (UPGMA) dendrogram was generated using MLST STs in 56 *C. coli* isolates based on allele numbers of seven MLST housekeeping genes. The image represents the distribution of aerotolerance levels, the proportion of 10 virulence genes, and antimicrobial resistance (AMR) of *C. coli* belonging to each MLST ST. The pie chart presents the distribution of aerotolerance levels (green, OS; blue, AT; red, HAT) among *C. coli* isolates. OS, oxygen-sensitive; AT, aerotolerant; HAT, hyper-aerotolerant.

In total, 18 MLST STs including the newly determined STs ST9575 and ST9867 were identified from 56 *C. coli* isolates. Six OS *C. coli* isolates belonged to five different STs. Twenty-two AT and 28 HAT *C. coli* isolates belonged to nine STs and 12 STs, respectively (Figure S2). The MST showed that all *C. coli* isolates were clustered closely. ST855 was the most common ST in this study (Figure 1 and Table S3). We then generated a UPGMA dendrogram showing the rates of 10 virulence genes, AMR, and aerotolerance levels (OS, AT, HAT) in *C. coli* isolates of each MLST genotype (Figure 2). Fifty percent of the *C. coli* isolates belonging to ST855 were HAT, resistant to at least two antimicrobials, and possessed a variety of virulence genes.

### 3.5. Genetic Relatedness of C. coli Isolates from Duck and Human Sources

**Figure 3:**
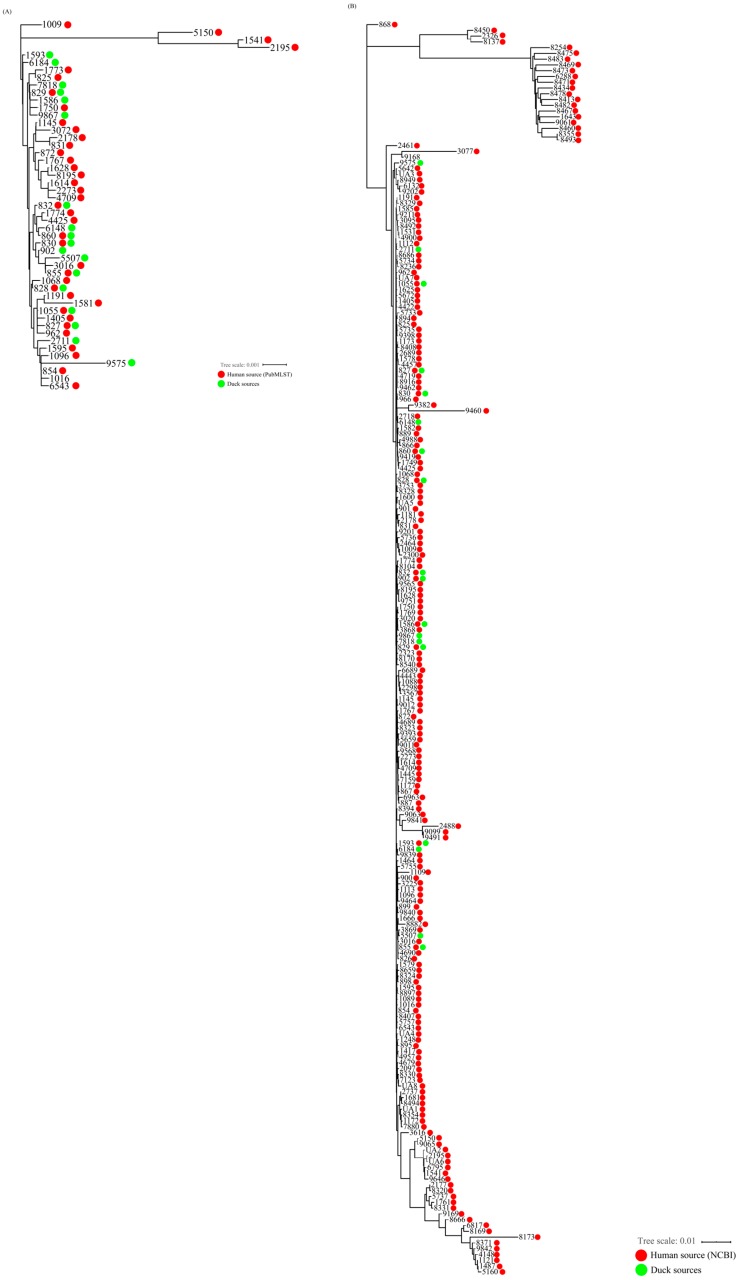
Genetic relatedness of *C. coli* isolates from duck sources in this study and those from a human source in (**A**) PubMLST and (**B**) NCBI. (A) Genetic relatedness of *C. coli* isolates from duck sources in this study and the human source (PubMLST). A neighbor-joining tree was constructed based on concatenated nucleotide sequences of seven housekeeping genes. The distances in the tree were computed using the maximum composite likelihood method. The tree represents the genetic relatedness between *C. coli* isolates from duck sources and those of a human source registered in PubMLST (http://pubmlst.org/campylobacter). Each color indicates a source (green, duck sources in this study; red, human source from PubMLST). For multi-locus sequence typing (MLST) sequence types (STs) of *C. coli* isolated from the human source in PubMLST, MLST STs that harbored at least 10 *C. coli* isolates were selected and used to analyze genetic relatedness to *C. coli* from this study. (**B**) Genetic relatedness between *C. coli* isolates from duck sources in this study and those of a human source (NCBI). In total, 217 MLST genotypes were identified from whole genome shotgun sequences (WGSs) of 848 *C. coli* isolates from human clinical cases registered in NCBI. A neighbor-joining tree and the distance in the tree were generated and calculated using the same manner as in (**A**). The tree represents the genetic relatedness of *C. coli* isolates from duck sources with those from the human source registered in NCBI (National Center for Biotechnology Information, US). Each color indicates a source (green, duck source in this study; red, human source from NCBI). UA (unassigned) refers to a genotype for which allelic numbers of seven MLST housekeeping genes (*aspA, glnA, gltA, glyA, pgm, tkt,* and *uncA*) were assigned but sequence type (ST) was not assigned.

**Figure 4:**
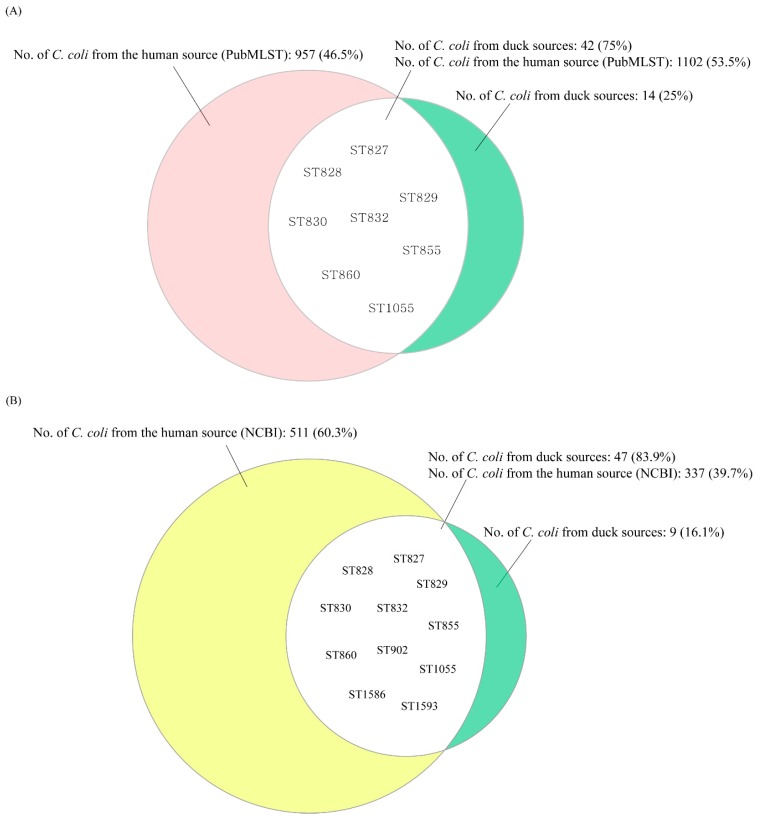
The proportion of *C. coli* isolates belonging to multi-locus sequence typing (MLST) sequence types (STs) shared between duck sources and the human source in (**A**) PubMLST and (**B**) NCBI. (**A**) The proportion of *C. coli* isolates belonging to MLST STs shared between the duck sources and human source (PubMLST). The proportions of *C. coli* isolates that belonged to the shared MLST STs were 75% and 53.5% in duck sources and the human source (PubMLST), respectively. The green section indicates the proportion of duck-derived *C. coli* belonging to the non-shared STs. The red section indicates the proportion of human-derived *C. coli* isolates belonging to the non-shared STs. The intersection (white) represents the proportion of *C. coli* isolates belonging to the eight MLST STs (ST827, ST828, ST829, ST830, ST832, ST855, ST860, and ST1055) shared between the duck sources and human source (PubMLST). (**B**) The proportion of *C. coli* isolates belonging to MLST STs shared between duck sources and the human source (NCBI). The proportions of *C. coli* isolates that belonged to the shared MLST STs were 83.9% and 39.7% in the duck sources and human source (NCBI), respectively. The green section indicates the proportion of duck-derived *C. coli* belonging to non-shared STs. The yellow section indicates the proportion of human-derived *C. coli* isolates belonging to non-shared STs. The intersection (white) represents the proportion of *C. coli* isolates belonging to the 11 MLST STs (ST827, ST828, ST829, ST830, ST832, ST855, ST860, ST902, ST1055, ST1586, and ST1593) shared between the duck sources and human source (NCBI).

We next analyzed the genetic relatedness of *C. coli* isolates from duck and human sources using 39 selected MLST STs, each of which contained at least 10 *C. coli* isolates (range: 11–689 isolates per ST; median: 17 isolates) from a human source (PubMLST, accessed on 28 January 2019) covering 2059 *C. coli* isolates. Analysis of the genetic relatedness of *C. coli* isolates from duck and human sources (PubMLST) based on the nucleotide sequences of seven MLST housekeeping genes revealed that eight STs (ST827, ST828, ST829, ST830, ST832, ST855, ST860, and ST1055) of 18 MLST STs in this study were shared with *C. coli* isolates from the human source (PubMLST) (Figure 3A). These eight STs contained 42 (75%) of the 56 *C. coli* isolates from duck sources in this study and 1102 (53.5%) of 2059 *C. coli* isolates from the human source (PubMLST) (Figure 4A). In addition, HAT *C. coli* isolates were predominant (57.1%) among the 42 isolates belonging to the eight STs that were shared with those of the human source (PubMLST). The proportion of HAT *C. coli* in these eight shared STs was significantly higher than that in the non-shared STs, whereas the proportion of OS followed the opposite trend (HAT vs OS: OR = 30.0, 95% CI (2.7, 328.6)) (Table 4).

We also used NCBI data, which included WGS data of approximately 850 human clinical *C. coli* isolates, to analyze the genetic relatedness between *C. coli* from duck sources in this study and those from humans in NCBI. We identified 217 MLST genotypes from the NCBI data and identified that 11 STs (ST827, ST828, ST829, ST830, ST832, ST855, ST860, ST902, ST1055, ST1586, ST1593) of 18 MLST STs in *C. coli* isolates from duck sources were shared with *C. coli* isolates from the human source (NCBI) (Figure 3B). The 11 STs comprised 47 (83.9%) of 56 *C. coli* isolates from duck sources and 337 (39.7%) of 848 *C. coli* isolates from the human source (NCBI) (Figure 4B). In addition, HAT *C. coli* isolates comprised the largest population (55.3%) among the 47 isolates of the 11 STs shared with the human source (NCBI). The proportion of HAT *C. coli* in the 11 shared STs was significantly higher than that in the non-shared STs, whereas the proportion of OS followed the opposite trend (HAT vs OS: OR = 65, 95% CI (4.9, 861.5)) (Table 5).

## 4. Discussion

Recently, concerns regarding the aerotolerance of *Campylobacter* have increased. Accordingly, the aerotolerance of *C. jejuni* isolates from retail chicken meat has been studied. However, the characteristics of duck-derived *Campylobacter*, especially aerotolerant *C. coli*, have not yet been studied. Here, we investigated the prevalence of HAT *C. coli* in duck sources (carcasses and meat) and its characteristics in order to assess potential impacts on public health. To the best of our knowledge, this is the first study to evaluate the potential impact of HAT *C. coli* on public health by investigating its prevalence in duck sources, analyzing its characteristics, and determining genetic relatedness with isolates from human sources.

We identified that almost half of the duck samples harbored *C. coli* (46.6%, 55/118). We found that the prevalence of *C. coli* was higher in ducks than in other livestock including chickens (26.4–31.8%) and cattle (7.7–15.0%) [19,34,35,36], indicating that ducks are a major reservoir of *C. coli* among food-producing animals. Furthermore, two different *C. coli* strains that were epidemiologically unrelated (phenotypically or genotypically) were identified in isolates from each of the six samples (10.9%) among the 55 *C. coli*-positive duck samples (Table S4).

HAT *C. coli* was predominant (50%) among the 56 *C. coli* strains used in this study. This finding indicates that HAT *C. coli* might survive longer in the external environment or under stress conditions at different stages of the manufacturing process, and may be transmitted to humans easily via diverse routes at various stages of the farm-to-table process including farming, processing, retail, and food preparation at homes or restaurants. We also found that *C. coli* strains from duck sources (HAT = 50%) in this study were more aerotolerant than *C. jejuni* from retail chicken meat (HAT = 5–35.7%) from previous studies [13,37]. In addition, *C. coli* strains from duck sources in this study may be more aerotolerant than *C. coli* from retail chicken meat (HAT = 11.1%) from a previous study [37]. These findings indicate that the potential transmission of *C. coli* to humans due to duck consumption might be higher than with chicken consumption.

To evaluate the potential impact of HAT *C. coli* on public health, we investigated the virulence potential and antimicrobial resistance of these strains. High prevalence of various virulence genes including *flaA*, *cadF*, *ceuE*, *cdtA,* and *wlaN*, which are involved in motility, adhesion, invasion, toxin production, and the expression of ganglioside mimics in Guillain-Barré syndrome, was observed in most HAT *C. coli*. This is similar to the results of a previous study on HAT *C. jejuni* in Canada [22], reporting that most HAT *C. jejuni* possessed various virulence genes related to toxin production (*cdtB*), cell adhesion (*cadF*, *peb1*, *pldA*), invasion (*ciaB*, *iam*), and colonization (*docA*) [38,39]; however, there was no significant difference from oxygen-sensitive isolates in this study. Moreover, almost all *C. coli* strains (98.2%, 55/56) in this study were resistant to quinolones including CIP, irrespective of aerotolerance levels, and strains with a high resistance to CIP (MIC value ≥ 32 μg/mL) were identified. Interestingly, most (77.8%) strains with a high resistance to CIP were identified as HAT, showing a positive tendency (*p* = 0.11) of HAT *C. coli* to be associated with high-level resistance to CIP. In addition, all HAT *C. coli* isolates showed resistance to at least two antimicrobials, presenting six AMR patterns including four MDR patterns (Table 3). HAT *C. coli* could pose a threat to public health due to its potential for virulence and antimicrobial resistance. This is of particular concern, as quinolone resistance in HAT *C. coli* might make it difficult to treat patients for campylobacteriosis, given that quinolones are usually used for clinical cases and especially severe cases [40,41]. The high proportion of quinolone resistance in this study can be explained by the widespread use of fluoroquinolones in South Korea and the characteristics of fluoroquinolone-resistant *Campylobacter*. In South Korea, quinolones were extensively used in the veterinary field until they were banned in July 2008 [42,43,44,45]. Furthermore, fluoroquinolone resistance can persist without antibiotic selection pressure, and fluoroquinolone-resistant *Campylobacter* was found to outcompete the majority of its clonally related fluoroquinolone-sensitive populations [46,47]. This indicates that fluoroquinolone resistance in *C. coli* may persist in *C. coli* populations even when fluoroquinolone use has ceased. Future studies are needed to understand the relationships among the mechanisms of aerotolerance, such as the expression of *katA*, *ahpC*, and *sodB* [48], the ferric uptake regulator (FUR) to cross-protect against oxidative stress [49], and the mechanisms of quinolone resistance such as threonine-to-isoleucine substitutions at amino acid 86 in the QRDR of GyrA in *Campylobacter* [50].

In total, 18 MLST STs, including two newly determined STs (ST9575 and ST9867) were clonally distributed in this study as determined using the MST method based on allelic numbers of MLST housekeeping genes (Figure 1). Of the 18 STs in this study, the most common ST was ST855, which was associated with a high proportion (50%) of HAT *C. coli* with resistance to at least two antimicrobials, as well as high proportions of virulence genes related to motility, adhesion, invasion, toxin production, and the expression of ganglioside mimics in Guillain-Barré syndrome (Figure 2). ST855 was reported as the most common genotype among *C. coli* isolates from patients with acute gastroenteritis in a previous study [51], and it was similarly identified as a common genotype from the human source registered in PubMLST (accessed on 28 January 2019). Based on these findings, we inferred that ST855 is possibly related to human *C. coli* infections, and that HAT *C. coli* of ST855 might pose a threat to public health based on antimicrobial resistance and virulence potential when transmitted to humans.

To analyze the genetic relatedness of *C. coli* from duck and human sources, 18 MLST STs from duck sources were compared in this study with a human database (PubMLST). Among them, eight STs were common to those of the human source and 75% of *C. coli* strains from duck sources belonged to the shared STs, indicating that ducks are possibly related to human *Campylobacter* infections’. Compared to OS *C. coli* from duck sources, HAT *C. coli* strains were more likely to be distributed among the shared STs (HAT vs OS: OR = 30.0, 95% CI (2.7, 328.6)) (Figure 3A and Figure 4A and Table 4). We further analyzed human clinical data from NCBI (WGS of 848 *C. coli* isolates) to determine their MLST STs. This analysis also showed that high proportions (83.9%) of *C. coli* strains from duck sources belonged to 11 STs common to those of the human source (Figure 3B and Figure 4B). In addition, compared to OS *C. coli* from duck sources, HAT *C. coli* strains accounted for a significant proportion of the shared STs (HAT vs OS: OR = 65, 95% CI (4.9, 861.5)) (Table 5). These results suggest that HAT *C. coli* from duck sources could be genetically related to the human source, and could be easily transmitted to humans through the food chain, although additional epidemiological data are required to confirm potential links between HAT *C. coli* from duck sources and human cases. This poses a threat to public health given its high prevalence, virulence potential, and antimicrobial resistance. Furthermore, *C. coli* belonging to the shared STs accounted for 53.5% of the *C. coli* registered in PubMLST data and for 39.7% of the isolates registered in the NCBI data. In addition, previous studies showed that most STs of the shared STs were related to human clinical cases [52,53,54]. From these results and those of a previous study reporting that the most clinical *C. jejuni* strains were HAT [55], it can be speculated that *C. coli* isolated from humans are possibly HAT.

## 5. Conclusions

HAT *C. coli* is prevalent in duck sources and survives longer on poultry meat, indicating that the possibility of HAT *C. coli* transmission to humans is relatively high. HAT *C. coli* possesses various virulence genes and shows resistance to antimicrobials at high levels, implying that these strains can be a threat to public health. Interestingly, we showed that most HAT *C. coli* from duck sources contained the same MLST genotypes as those of the human source (PubMLST data, NCBI data), suggesting that HAT *C. coli* could be genetically related to human infections. This study provides information regarding the impact of HAT *C. coli* strains on public health due to their aerotolerance, virulence, and antimicrobial resistance. This will assist in improving control strategies to reduce the farm-to-table transmission of HAT *C. coli* to humans.

## Figures and Tables

**Table 1:** Prevalence of virulence genes in *Campylobacter coli* isolates according to aerotolerance levels.

Aerotolerance Levels	No. Isolates	Virulence Genes									
		*flaA*	*flhB*	*cadF*	*pldA*	*iamA*	*ceuE*	*cdtA*	*wlaN*	*hcp*	*virB11*
OS	6	83.3%	0%	100%	0%	0%	83.3%	100%	83.3%	16.7%	0%
AT	22	100%	0%	95.5%	0%	4.5%	90.9%	95.5%	100%	13.6%	0%
HAT	28	75%	3.6%	89.3%	7.1%	14.3%	78.6%	96.4%	89.3%	3.6%	7.1%
Total	56	85.7%	1.8%	92.9%	3.6%	8.9%	83.9%	96.4%	92.9%	8.9%	3.6%

OS, oxygen-sensitive; AT, aerotolerance; HAT, hyper-aerotolerance.

**Table 2:** Distribution of minimum inhibitory concentration (MIC) values (μg/mL) for *C. coli* isolates according to aerotolerance levels.

Agents	Aerotolerance Levels	0.03	0.06	0.12	0.25	0.5	1	2	4	6	8	16	32	64	>64
**ERY**	**OS**					2	2								2
	**AT**			3	7	6	3	2						1	
	**HAT**			1	7	5	8	5						1	1
**CHL**	**OS**							2	3		1				
	**AT**							17	5						
	**HAT**							15	9		4				
**CIP**	**OS**				1						1	4			
	**AT**										11	9	2		
	**HAT**								2		10	9	6	1	
**TET**	**OS**											2		1	3
	**AT**				4	1					1		1	5	10
	**HAT**				4	3	1	1						5	14
**TEL**	**OS**						2	2			2				
	**AT**			3	5	5	4	1	3		1				
	**HAT**				6	7	4	4	4		3				
**GEN**	**OS**				2	2							2		
	**AT**				4	15	2						1		
	**HAT**				1	24	2						1		
**AZI**	**OS**		3	1											2
	**AT**	3	13	5											1
	**HAT**	2	13	10	1									1	1
**STR**	**OS**						2	1				3			
	**AT**						4	15				3			
	**HAT**						7	13	2			6			
**NAL**	**OS**										1			5	
	**AT**													18	4
	**HAT**													22	6

Green, MIC values for resistance to each antimicrobial agent; orange, MIC values for high-level resistance to each antimicrobial agent; OS, oxygen-sensitive; AT, aerotolerance; HAT, hyper-aerotolerance; ERY, erythromycin; CHL, chloramphenicol; CIP, ciprofloxacin; TET, tetracycline; TEL, telithromycin; GEN, gentamicin; AZI, azithromycin; STR, streptomycin; NAL, nalidixic acid.

**Table 3:** Antimicrobial resistance (AMR) patterns according to aerotolerance levels in *C. coli* isolates.

AMR Patterns	No. of Isolates	OS	AT	HAT
TET	1	1	0	0
CIP-NAL	11	0	4	7
CIP-NAL-TET	30	2	15	13
CIP-NAL-TET-STR*	8	1	2	5
CIP-NAL-TET-STR-TEL*	1	0	0	1
CIP-NAL-STR-ERY-TEL-AZI-GEN*	2	0	1	1
CIP-NAL-TET-STR-ERY-TEL-AZI*	1	0	0	1
CIP-NAL-TET-STR-ERY-TEL-AZI-GEN*	2	2	0	0

* MDR patterns; OS, oxygen-sensitive; AT, aerotolerance; HAT, hyper-aerotolerance; ERY, erythromycin; CIP, ciprofloxacin; TET, tetracycline; TEL, telithromycin; GEN, gentamicin; AZI, azithromycin; STR, streptomycin; NAL, nalidixic acid.

**Table 4:** Comparison of the proportion of shared multi-locus sequence typing (MLST) sequence types (STs) with those of the human source (PubMLST) and non-shared STs according to aerotolerance levels of *C. coli*.

Aerotolerance Levels	Eight Shared STs ^*^	Not-Shared STs ^**^	Odds	OR (95% CI) ^†^
				HAT vs OS
Oxygen-sensitive (OS)	**1 (2.4%)**	**5 (35.7%)**	0.2	30 (2.7, 328.6)
Aerotolerant (AT)	17 (40.5%)	5 (35.7%)	3.4	
Hyper-aerotolerant (HAT)	24 (57.1%)	4 (28.6%)	6	
Total	42	14		
	56			

^*^ Eight STs (MLST sequence types ST827, ST828, ST829, ST830, ST832, ST855, ST860, and ST1055) shared between duck sources and the human source (PubMLST). ^**^ MLST STs that were not shared between duck sources and the human source (PubMLST). ^†^ OR (95% CI).

**Table 5:** Comparison of the proportion of shared multi-locus sequence typing (MLST) sequence types (STs) with those of the human source (NCBI) and non-shared STs according to aerotolerance levels of *C. coli.*

Aerotolerance Levels	Eleven Shared STs ^*^	Not-Shared STs ^**^	Odds	OR (95% CI) ^†^
				HAT vs OS
Oxygen-sensitive (OS)	1 (2.1%)	5 (55.6%)	0.2	65 (4.9, 861.5)
Aerotolerant (AT)	20 (42.6%)	2 (22.2%)	10	
Hyper-aerotolerant (HAT)	26 (55.3%)	2 (22.2%)	13	
Total	47	9		
	56			

^*^ Eleven STs (MLST sequence types ST827, ST828, ST829, ST830, ST832, ST855, ST860, ST902, ST1055, ST1586, and ST1593) shared between duck sources and the human source (NCBI). ^**^ MLST STs that were not shared between duck sources and the human source (NCBI). ^†^ OR (95% CI).

## Supplementary Materials

Supplementary materials can be found at https://www.mdpi.com/2076-2607/7/11/579/s1.

## References

[1] Alfredson D.A., Korolik V. (2007). Antibiotic resistance and resistance mechanisms in *Campylobacter jejuni* and *Campylobacter coli*. FEMS Microbiol. Lett..

[2] Kaakoush N.O., Castaño-Rodríguez N., Mitchell H.M., Man S.M. (2015). Global epidemiology of Campylobacter infection. Clin. Microbiol. Rev..

[3] Azrad M., Tkhawkho L., Isakovich N., Nitzan O., Peretz A. (2018). Antimicrobial susceptibility of *Campylobacter jejuni* and *Campylobacter coli*: Comparison between Etest and a broth dilution method. Ann. Clin. Microbiol. Antimicrob..

[4] Datta S., Niwa H., Itoh K. (2003). Prevalence of 11 pathogenic genes of *Campylobacter jejuni* by PCR in strains isolated from humans, poultry meat and broiler and bovine faeces. J. Med. Microbiol..

[5] Sheppard S.K., Maiden M.C. (2015). The evolution of *Campylobacter jejuni* and *Campylobacter coli*. Cold Spring Harb. Perspect. Biol..

[6] An J.-U., Ho H., Kim J., Kim W.-H., Kim J., Lee S., Mun S.-H., Guk J.-H., Hong S., Cho S. (2018). Dairy cattle, a potential reservoir of human campylobacteriosis: Epidemiological and molecular characterization of *Campylobacter jejuni* from cattle farms. Front. Microbiol..

[7] Gargiulo A., Sensale M., Marzocco L., Fioretti A., Menna L.F., Dipineto L. (2011). *Campylobacter jejuni*, *Campylobacter coli*, and cytolethal distending toxin (CDT) genes in common teals (Anas crecca). Vet. Microbiol..

[8] Giacomelli M., Salata C., Martini M., Montesissa C., Piccirillo A. (2014). Antimicrobial resistance of *Campylobacter jejuni* and *Campylobacter coli* from poultry in Italy. Microb. Drug Resist..

[9] Sahin O., Kassem I.I., Shen Z., Lin J., Rajashekara G., Zhang Q. (2015). Campylobacter in Poultry: Ecology and Potential Interventions. Avian Dis..

[10] Kassem I.I., Kehinde O., Kumar A., Rajashekara G. (2017). Antimicrobial-Resistant Campylobacter in Organically and Conventionally Raised Layer Chickens. Foodborne Pathog. Dis..

[11] Kaakoush N.O., Miller W.G., De Reuse H., Mendz G.L. (2007). Oxygen requirement and tolerance of *Campylobacter jejuni*. Res. Microbiol..

[12] Rodrigues R.C., Pocheron A.-L., Hernould M., Haddad N., Tresse O., Cappelier J.-M. (2015). Description of *Campylobacter jejuni* Bf, an atypical aero-tolerant strain. Gut Pathog..

[13] Oh E., McMullen L., Jeon B. (2015). High Prevalence of Hyper-Aerotolerant *Campylobacter jejuni* in Retail Poultry with Potential Implication in Human Infection. Front. Microbiol..

[14] Silva J., Leite D., Fernandes M., Mena C., Gibbs P.A., Teixeira P. (2011). *Campylobacter* spp. as a foodborne pathogen: A review. Front. Microbiol..

[15] Grant A., Hashem F., Parveen S. (2016). Salmonella and Campylobacter: Antimicrobial resistance and bacteriophage control in poultry. Food Microbiol..

[16] Unicomb L.E., Fullerton K.E., Kirk M.D., Stafford R.J. (2009). Outbreaks of campylobacteriosis in Australia, 2001 to 2006. Foodborne Pathog. Dis..

[17] Wei B., Cha S.Y., Kang M., Roh J.H., Seo H.S., Yoon R.H., Jang H.K. (2014). Antimicrobial susceptibility profiles and molecular typing of *Campylobacter jejuni* and *Campylobacter coli* isolates from ducks in South Korea. Appl. Environ. Microbiol..

[18] Chon J.W., Lee S.K., Yoon Y., Yoon K.S., Kwak H.S., Joo I.S., Seo K.H. (2018). Quantitative prevalence and characterization of Campylobacter from chicken and duck carcasses from poultry slaughterhouses in South Korea. Poult. Sci..

[19] Little C.L., Richardson J.F., Owen R.J., de Pinna E., Threlfall E.J. (2008). Prevalence, characterisation and antimicrobial resistance of Campylobacter and Salmonella in raw poultrymeat in the UK, 2003–2005. Int. J. Environ. Health Res..

[20] Nor Faiza S., Saleha A.A., Jalila A., Fauziah N. (2013). Occurrence of Campylobacter and Salmonella in ducks and duck eggs in Selangor, Malaysia. Trop. Biomed..

[21] Hamed E.A., AbdelRahman M.A., Shalaby A.G., Morsy M.M., Nasef S.A. (2016). Antibiotic resistance and polymorphism in the quinolone resistance-determining region of *Campylobacter* spp. isolated from 1-day-old ducklings. Vet. J..

[22] Oh E., McMullen L.M., Chui L., Jeon B. (2017). Differential Survival of Hyper-Aerotolerant *Campylobacter jejuni* under Different Gas Conditions. Front. Microbiol..

[23] Konkel M.E., Gray S.A., Kim B.J., Garvis S.G., Yoon J. (1999). Identification of the Enteropathogens *Campylobacter jejuni* and *Campylobacter coli* Based on the cadF Virulence Gene and Its Product. J. Clin. Microbiol..

[24] Bacon D.J., Alm R.A., Burr D.H., Hu L., Kopecko D.J., Ewing C.P., Guerry P. (2000). Involvement of a plasmid in virulence of *Campylobacter jejuni* 81-176. Infect. Immun..

[25] Linton D., Gilbert M., Hitchen P.G., Dell A., Morris H.R., Wakarchuk W.W., Gregson N.A., Wren B.W. (2000). Phase variation of a β-1, 3 galactosyltransferase involved in generation of the ganglioside GM1-like lipo-oligosaccharide of *Campylobacter jejuni*. Mol. Microbiol..

[26] Corcionivoschi N., Gundogdu O., Moran L., Kelly C., Scates P., Stef L., Cean A., Wren B., Dorrell N., Madden R.H. (2015). Virulence characteristics of hcp+ *Campylobacter jejuni* and *Campylobacter coli* isolates from retail chicken. Gut Pathog..

[27] Koolman L., Whyte P., Burgess C., Bolton D. (2015). Distribution of virulence-associated genes in a selection of Campylobacter isolates. Foodborne Pathog. Dis..

[28] Guerry P. (2007). Campylobacter flagella: Not just for motility. Trends Microbiol..

[29] Dasti J.I., Tareen A.M., Lugert R., Zautner A.E., Gross U. (2010). *Campylobacter jejuni*: A brief overview on pathogenicity-associated factors and disease-mediating mechanisms. Int. J. Med. Microbiol. IJMM.

[30] European Committee on Antimicrobial Susceptibility Testing Clinical Breakpoints, Epidemiological Cut-Off (ECOFF) Values and EUCAST Disk Diffusion Methodology for Campylobacter jejuni and Campylobacter coli. http://www.eucast.org/fileadmin/src/media/PDFs/EUCAST_files/Consultation/Campylobacter_wide_consultation_August_2012.pdf.

[31] Segreti J., Gootz T.D., Goodman L.J., Parkhurst G.W., Quinn J.P., Martin B.A., Trenholme G.M. (1992). High-level quinolone resistance in clinical isolates of *Campylobacter jejuni*. J. Infect. Dis..

[32] Engberg J., Aarestrup F.M., Taylor D.E., Gerner-Smidt P., Nachamkin I. (2001). Quinolone and macrolide resistance in *Campylobacter jejuni* and *C. coli*: Resistance mechanisms and trends in human isolates. Emerg. Infect. Dis..

[33] Magiorakos A.P., Srinivasan A., Carey R., Carmeli Y., Falagas M., Giske C., Harbarth S., Hindler J., Kahlmeter G., Olsson-Liljequist B. (2012). Multidrug-resistant, extensively drug-resistant and pandrug-resistant bacteria: An international expert proposal for interim standard definitions for acquired resistance. Clin. Microbiol. Infect..

[34] Bae W., Kaya K.N., Hancock D.D., Call D.R., Park Y.H., Besser T.E. (2005). Prevalence and antimicrobial resistance of thermophilic *Campylobacter* spp. from cattle farms in Washington State. Appl. Environ. Microbiol..

[35] Kang Y.-S., Cho Y.-S., Yoon S.-K., Yu M.-A., Kim C.-M., Lee J.-O., Pyun Y.-R. (2006). Prevalence and antimicrobial resistance of *Campylobacter jejuni* and *Campylobacter coli* isolated from raw chicken meat and human stools in Korea. J. Food Prot..

[36] Tang Y., Sahin O., Pavlovic N., LeJeune J., Carlson J., Wu Z., Dai L., Zhang Q. (2017). Rising fluoroquinolone resistance in Campylobacter isolated from feedlot cattle in the United States. Sci. Rep..

[37] Karki A.B., Marasini D., Oakey C.K., Mar K., Fakhr M.K. (2018). *Campylobacter coli* from Retail Liver and Meat Products is More Aerotolerant than *Campylobacter jejuni*. Front. Microbiol..

[38] Bolton D.J. (2015). Campylobacter virulence and survival factors. Food Microbiol..

[39] Hendrixson D.R., DiRita V.J. (2004). Identification of *Campylobacter jejuni* genes involved in commensal colonization of the chick gastrointestinal tract. Mol. Microbiol..

[40] Sato K., Bartlett P., Kaneene J., Downes F. (2004). Comparison of prevalence and antimicrobial susceptibilities of Campylobacter spp. isolates from organic and conventional dairy herds in Wisconsin. Appl. Environ. Microbiol..

[41] Payot S., Bolla J.-M., Corcoran D., Fanning S., Mégraud F., Zhang Q. (2006). Mechanisms of fluoroquinolone and macrolide resistance in *Campylobacter* spp.. Microbes Infect..

[42] Ku B.K., Kim H.J., Lee Y.J., Kim Y.I., Choi J.S., Park M.Y., Kwon J.W., Nam H.M., Kim Y.H., Jung S.C. (2011). Genetic characterization and antimicrobial susceptibility of *Campylobacter* spp. isolated from domestic and imported chicken meats and humans in Korea. Foodborne Pathog. Dis..

[43] Wimalasena S., De Silva B.C.J., Hossain S., Pathirana H., Heo G.J. (2017). Prevalence and characterisation of quinolone resistance genes in Aeromonas spp. isolated from pet turtles in South Korea. J. Glob. Antimicrob. Resist..

[44] Na S.H., Moon D.C., Choi M.J., Oh S.J., Jung D.Y., Sung E.J., Kang H.Y., Hyun B.H., Lim S.K. (2019). Antimicrobial Resistance and Molecular Characterization of Extended-Spectrum beta-Lactamase-Producing *Escherichia coli* Isolated from Ducks in South Korea. Foodborne Pathog. Dis..

[45] Na S.H., Moon D.C., Choi M.J., Oh S.J., Jung D.Y., Kang H.Y., Hyun B.H., Lim S.K. (2019). Detection of oxazolidinone and phenicol resistant enterococcal isolates from duck feces and carcasses. Int. J. Food Microbiol..

[46] Luo N., Pereira S., Sahin O., Lin J., Huang S., Michel L., Zhang Q. (2005). Enhanced in vivo fitness of fluoroquinolone-resistant *Campylobacter jejuni* in the absence of antibiotic selection pressure. Proc. Natl. Acad. Sci. USA.

[47] Luangtongkum T., Jeon B., Han J., Plummer P., Logue C.M., Zhang Q. (2009). Antibiotic resistance in Campylobacter: Emergence, transmission and persistence. Future Microbiol..

[48] Oh E., McMullen L., Jeon B. (2015). Impact of oxidative stress defense on bacterial survival and morphological change in *Campylobacter jejuni* under aerobic conditions. Front. Microbiol..

[49] Askoura M., Sarvan S., Couture J.F., Stintzi A. (2016). The *Campylobacter jejuni* Ferric Uptake Regulator Promotes Acid Survival and Cross-Protection against Oxidative Stress. Infect. Immun..

[50] Elhadidy M., Miller W.G., Arguello H., Alvarez-Ordonez A., Dierick K., Botteldoorn N. (2019). Molecular epidemiology and antimicrobial resistance mechanisms of *Campylobacter coli* from diarrhoeal patients and broiler carcasses in Belgium. Transbound. Emerg. Dis..

[51] Duarte A., Santos A., Manageiro V., Martins A., Fraqueza M.J., Canica M., Domingues F.C., Oleastro M. (2014). Human, food and animal *Campylobacter* spp. isolated in Portugal: High genetic diversity and antibiotic resistance rates. Int. J. Antimicrob. Agents.

[52] Litrup E., Torpdahl M., Nielsen E. (2007). Multilocus sequence typing performed on *Campylobacter coli* isolates from humans, broilers, pigs and cattle originating in Denmark. J. Appl. Microbiol..

[53] Niederer L., Kuhnert P., Egger R., Buttner S., Hachler H., Korczak B.M. (2012). Genotypes and antibiotic resistances of *Campylobacter jejuni* and *Campylobacter coli* isolates from domestic and travel-associated human cases. Appl. Environ. Microbiol..

[54] Asakura H., Sakata J., Nakamura H., Yamamoto S., Murakami S. (2019). Phylogenetic Diversity and Antimicrobial Resistance of *Campylobacter coli* from Humans and Animals in Japan. Microbes Environ..

[55] Oh E., Chui L., Bae J., Li V., Ma A., Mutschall S.K., Taboada E.N., McMullen L.M., Jeon B. (2018). Frequent Implication of Multistress-Tolerant *Campylobacter jejuni* in Human Infections. Emerg. Infect. Dis..

