# Supplementary material for: Hyper-Aerotolerant Campylobacter coli from Duck Sources and Its Potential Threat to Public Health: Virulence, Antimicrobial Resistance, and Genetic Relatedness
Source: Microorganisms. 2019 Nov 19;7(11):579. doi: 10.3390/microorganisms7110579 (PMC6920863; doi:10.3390/microorganisms7110579)
Supplement: Supplementary file 1 [file microorganisms-07-00579-s001.pdf]

Table S1. Primers for identification of *Campylobacter coli* and its virulence genes.

Target Primers		Primer Sequence	Product (bp)	Reference	Annealing Temperature (°C)
Campylobacter 16s rDNA	F	GGA TGA CAC TTT TCG GAG C	816	[1]	57
	R	CAT TGT AGC ACG TGT GTC			
ask	F	GGT ATG ATT TCT ACA AAG CGA G	502	[1]	57
	R	ATA AAA GAC TAT CGT CGC GTG			
flaA	F	GGATTTCGTATTAACACAAATGGTGC	1722	[2]	55
	R	CTGTAGTAATCTTAAAACATTTTG			
flhB	F	TGGCAGGCGAAGATCAAGAA	549	[3]	55
	R	GCCAAGTAAGCTGTGCAACC			
cadF	F	TTGAAGGTAATTTAGATATG	400	[4]	45
	R	CTAATACCTAAAGTTGAAAC			
pldA	F	AAGCTTATGCGTTTTT	913	[5]	45
	R	TATAAGGCTTTCTCCA			
iamA	F	GCACAAAATATATCATTACAA	518	[6]	52
	R	TTCACGACTACTATGAGG			
ceuE	F	ATGAAAAAATATTTAGTTTTTGCA	984	[7]	57
	R	ATTTTATTATTTGTAGCAGCG			
cdtA	F	GGAAATTGGATTTGGGGCTATACT	165	[8,9]	42
	R	ATCACAAGGATAATGGACAAT			
wlaN	F	TGCTGGGTATACAAAGGTTGTG	330	[6]	60
	R	AATTTTGGATATGGGTGGGG			
hcp	F	CAAGCGGTGCATCTACTGAA	463	[10,11]	55
	R	TAAGCTTTGCCCTCTCTCCA			
virB11	F	GAACAGGAAGTGGAAAAACTAGC	708	[12]	50
	R	TTCCGCATTGGGCTATATG			

Table S2. Assembly accession numbers of whole genome shotgun sequences of *Campylobacter coli* clinical isolates registered in NCBI (National Center for Biotechnology Information, U.S. https://www.ncbi.nlm.nih.gov/).

NCBI Assembly Accession					
GCA_001418825.1	GCA_005240505.1	GCA_008238345.1	GCA_008227945.1	GCA_008232045.1	GCA_008348245.1
GCA_001418845.1	GCA_005223415.1	GCA_008136225.1	GCA_008230345.1	GCA_008234125.1	GCA_008348865.1
GCA_001419005.1	GCA_005262775.1	GCA_008140245.1	GCA_008349265.1	GCA_008238885.1	GCA_008205925.1
GCA_001419325.1	GCA_006349395.1	GCA_008275885.1	GCA_008237265.1	GCA_008133885.1	GCA_008219945.1
GCA_001419395.1	GCA_006349485.1	GCA_008276345.1	GCA_008237785.1	GCA_008161765.1	GCA_008230225.1
GCA_001419455.1	GCA_006370415.1	GCA_008282475.1	GCA_008344325.1	GCA_008161825.1	GCA_008311195.1
GCA_001419265.1	GCA_001224065.1	GCA_008290875.1	GCA_008346165.1	GCA_008137425.1	GCA_007931265.1
GCA_001419275.1	GCA_001230785.1	GCA_008290255.1	GCA_007902325.1	GCA_008137745.1	GCA_007967555.1
GCA_001488335.1	GCA_001231965.1	GCA_008291075.1	GCA_008126785.1	GCA_008135625.1	GCA_008144645.1
GCA_001490695.1	GCA_001232025.1	GCA_008292035.1	GCA_008245395.1	GCA_008136785.1	GCA_007901995.1
GCA_001490735.1	GCA_001237325.1	GCA_008311505.1	GCA_008230665.1	GCA_008138145.1	GCA_007927505.1
GCA_001492375.1	GCA_000314385.1	GCA_008334985.1	GCA_007898445.1	GCA_008139445.1	GCA_007928865.1
GCA_001496435.1	GCA_005264085.1	GCA_008344625.1	GCA_007920875.1	GCA_008143265.1	GCA_007937725.1
GCA_001497295.1	GCA_005225295.1	GCA_008346045.1	GCA_007929025.1	GCA_008141705.1	GCA_007945985.1
GCA_001497695.1	GCA_005232455.1	GCA_001234385.1	GCA_007933205.1	GCA_008141895.1	GCA_007947465.1
GCA_001497935.1	GCA_005228735.1	GCA_001235345.1	GCA_007933605.1	GCA_008142405.1	GCA_008140105.1
GCA_001498295.1	GCA_005243125.1	GCA_001229965.1	GCA_007934965.1	GCA_008142525.1	GCA_007942565.1
GCA_001487305.1	GCA_005241725.1	GCA_001230745.1	GCA_007935005.1	GCA_008144325.1	GCA_008253645.1
GCA_001487495.1	GCA_005230035.1	GCA_001226185.1	GCA_007941005.1	GCA_008145005.1	GCA_008345025.1
GCA_001488095.1	GCA_001223945.1	GCA_001237165.1	GCA_007941325.1	GCA_008151245.1	GCA_007983945.1
GCA_001488215.1	GCA_001225145.1	GCA_001234805.1	GCA_007942765.1	GCA_008154985.1	GCA_007986765.1
GCA_001488495.1	GCA_001225605.1	GCA_001237925.1	GCA_007959185.1	GCA_008154965.1	GCA_007981825.1
GCA_001489295.1	GCA_001226985.1	GCA_001226885.1	GCA_007967945.1	GCA_008126625.1	GCA_007987665.1
GCA_001489415.1	GCA_001228985.1	GCA_001228705.1	GCA_007970865.1	GCA_008127325.1	GCA_008216285.1
GCA_001489735.1	GCA_001229345.1	GCA_001239125.1	GCA_007970125.1	GCA_008127225.1	GCA_008338085.1
GCA_001489855.1	GCA_001229385.1	GCA_001239665.1	GCA_007983565.1	GCA_008126825.1	GCA_007899165.1
GCA_001489955.1	GCA_001230805.1	GCA_001227865.1	GCA_008207425.1	GCA_008246465.1	GCA_008279105.1
GCA_001490555.1	GCA_001231485.1	GCA_007895495.1	GCA_008207785.1	GCA_008276425.1	GCA_008139045.1
GCA_001492655.1	GCA_001232685.1	GCA_007920715.1	GCA_008216885.1	GCA_008279895.1	GCA_008139565.1
GCA_001492875.1	GCA_001233705.1	GCA_007927385.1	GCA_008218735.1	GCA_008284635.1	GCA_008141925.1
GCA_001493555.1	GCA_001235285.1	GCA_007929325.1	GCA_008218965.1	GCA_008282575.1	GCA_008126045.1
GCA_001493975.1	GCA_001236625.1	GCA_007930905.1	GCA_008223005.1	GCA_008293615.1	GCA_008038265.1
GCA_001495035.1	GCA_001237025.1	GCA_007931005.1	GCA_008223065.1	GCA_008294035.1	GCA_007948425.1
GCA_001495735.1	GCA_001237285.1	GCA_007931275.1	GCA_008229505.1	GCA_008295415.1	GCA_008218595.1

GCA_001495975.1	GCA_001239065.1	GCA_007935445.1	GCA_008229585.1	GCA_008336105.1	GCA_008249325.1
GCA_001496095.1	GCA_001239285.1	GCA_007940845.1	GCA_008229645.1	GCA_008335785.1	GCA_008136025.1
GCA_001498275.1	GCA_002178515.1	GCA_007937705.1	GCA_008232125.1	GCA_008338005.1	GCA_008232865.1
GCA_001498515.1	GCA_005223715.1	GCA_007944905.1	GCA_008234435.1	GCA_008340445.1	GCA_008235345.1
GCA_001498655.1	GCA_005227155.1	GCA_007942145.1	GCA_008235585.1	GCA_008341065.1	GCA_007949005.1
GCA_001419145.1	GCA_005227315.1	GCA_007943525.1	GCA_008239555.1	GCA_008345185.1	GCA_007951705.1
GCA_001419225.1	GCA_005223835.1	GCA_007943865.1	GCA_008133825.1	GCA_008346605.1	GCA_007897765.1
GCA_001419345.1	GCA_005224475.1	GCA_007944325.1	GCA_008134785.1	GCA_008346965.1	GCA_007899285.1
GCA_001419355.1	GCA_005240745.1	GCA_007945965.1	GCA_008162005.1	GCA_008347225.1	GCA_007984765.1
GCA_001419385.1	GCA_005224635.1	GCA_007947085.1	GCA_008162105.1	GCA_008347145.1	GCA_007988345.1
GCA_001419525.1	GCA_005224915.1	GCA_007944505.1	GCA_008135305.1	GCA_008348385.1	GCA_008215625.1
GCA_001419015.1	GCA_005241325.1	GCA_007948205.1	GCA_008138165.1	GCA_008348885.1	GCA_007987285.1
GCA_001491255.1	GCA_005242285.1	GCA_007948285.1	GCA_008141085.1	GCA_008354645.1	GCA_008217345.1
GCA_001491935.1	GCA_005226195.1	GCA_007948485.1	GCA_008143585.1	GCA_007965065.1	GCA_008251125.1
GCA_001493815.1	GCA_005226535.1	GCA_007949445.1	GCA_008142785.1	GCA_007985565.1	GCA_008279885.1
GCA_001494915.1	GCA_005225255.1	GCA_007949225.1	GCA_008143165.1	GCA_007988525.1	GCA_008249225.1
GCA_001495055.1	GCA_005264255.1	GCA_007963765.1	GCA_008154845.1	GCA_008038305.1	GCA_008343205.1
GCA_001495635.1	GCA_005226735.1	GCA_007952725.1	GCA_008156845.1	GCA_008209815.1	GCA_007933945.1
GCA_001498315.1	GCA_005241605.1	GCA_007966785.1	GCA_008126905.1	GCA_008216625.1	GCA_007944985.1
GCA_001492535.1	GCA_005242065.1	GCA_007956085.1	GCA_008128755.1	GCA_008231765.1	GCA_008234595.1
GCA_001492275.1	GCA_005232295.1	GCA_007959085.1	GCA_008128885.1	GCA_008232465.1	GCA_007948085.1
GCA_001498475.1	GCA_005241045.1	GCA_007981685.1	GCA_008247935.1	GCA_008233065.1	GCA_008226225.1
GCA_001490535.1	GCA_005242445.1	GCA_007983465.1	GCA_008250965.1	GCA_008234985.1	GCA_007952825.1
GCA_001487325.1	GCA_005261965.1	GCA_008038165.1	GCA_008253845.1	GCA_008234025.1	GCA_008282555.1
GCA_001493495.1	GCA_005240845.1	GCA_007986965.1	GCA_008254165.1	GCA_008237245.1	GCA_008291035.1
GCA_001491835.1	GCA_005241965.1	GCA_007987165.1	GCA_008279945.1	GCA_008157885.1	GCA_007949345.1
GCA_001492455.1	GCA_005262235.1	GCA_008207845.1	GCA_009254325.1	GCA_008141245.1	GCA_008239525.1
GCA_001498535.1	GCA_005229215.1	GCA_008209025.1	GCA_008282335.1	GCA_008281195.1	GCA_008135025.1
GCA_001490515.1	GCA_005228595.1	GCA_008209785.1	GCA_008281495.1	GCA_008283895.1	GCA_008140025.1
GCA_001488615.1	GCA_005228255.1	GCA_008218205.1	GCA_008286375.1	GCA_008291335.1	GCA_008132725.1
GCA_001493395.1	GCA_005243585.1	GCA_008225945.1	GCA_008285275.1	GCA_008294935.1	GCA_008152005.1
GCA_001488975.1	GCA_005243305.1	GCA_008227765.1	GCA_008294815.1	GCA_008339325.1	GCA_007947705.1
GCA_001490415.1	GCA_005262955.1	GCA_008230165.1	GCA_008305035.1	GCA_008347045.1	GCA_008205245.1
GCA_001498195.1	GCA_005263155.1	GCA_008232665.1	GCA_008310335.1	GCA_007952205.1	GCA_008209725.1
GCA_001495235.1	GCA_005263315.1	GCA_008234725.1	GCA_008310555.1	GCA_007981845.1	GCA_008280405.1
GCA_001497095.1	GCA_005232495.1	GCA_008235745.1	GCA_008332365.1	GCA_008230785.1	GCA_008341405.1
GCA_001494475.1	GCA_006349425.1	GCA_008235985.1	GCA_008333265.1	GCA_008231165.1	GCA_008346525.1
GCA_001496515.1	GCA_001223685.1	GCA_008236185.1	GCA_008338505.1	GCA_008235605.1	GCA_008345085.1

GCA_001496835.1	GCA_900446325.1	GCA_008237015.1	GCA_008341045.1	GCA_008133405.1	GCA_008344175.1
GCA_001488035.1	GCA_001228905.1	GCA_008131815.1	GCA_008341125.1	GCA_008144885.1	GCA_007948565.1
GCA_001492195.1	GCA_001236965.1	GCA_008132105.1	GCA_008341185.1	GCA_008145145.1	GCA_007898265.1
GCA_001494535.1	GCA_008011655.1	GCA_008132945.1	GCA_008341645.1	GCA_008284045.1	GCA_008206565.1
GCA_001499575.1	GCA_005223775.1	GCA_008133605.1	GCA_008341685.1	GCA_008292115.1	GCA_007964045.1
GCA_001491135.1	GCA_005224975.1	GCA_008133585.1	GCA_008343515.1	GCA_008281935.1	GCA_007952445.1
GCA_001492975.1	GCA_005229375.1	GCA_008132425.1	GCA_008345905.1	GCA_008233325.1	GCA_007972205.1
GCA_001491215.1	GCA_005243185.1	GCA_008134065.1	GCA_008346845.1	GCA_008228105.1	GCA_008250605.1
GCA_001495375.1	GCA_006347295.1	GCA_008135985.1	GCA_008769235.1	GCA_008237305.1	GCA_008225485.1
GCA_001499475.1	GCA_006348765.1	GCA_008139385.1	GCA_009175245.1	GCA_008137605.1	GCA_008228825.1
GCA_001493115.1	GCA_006349415.1	GCA_008141425.1	GCA_008014255.1	GCA_008135545.1	GCA_008229895.1
GCA_001491055.1	GCA_006349495.1	GCA_008142065.1	GCA_008222885.1	GCA_008332005.1	GCA_008218885.1
GCA_001496295.1	GCA_006370375.1	GCA_008142145.1	GCA_007928475.1	GCA_008278505.1	GCA_008235145.1
GCA_001490235.1	GCA_006370435.1	GCA_008144965.1	GCA_008225465.1	GCA_008290475.1	GCA_008239745.1
GCA_001496735.1	GCA_005226755.1	GCA_008151185.1	GCA_008229605.1	GCA_008290195.1	GCA_008218425.1
GCA_001491755.1	GCA_006349385.1	GCA_008151755.1	GCA_008289535.1	GCA_008305675.1	GCA_008226805.1
GCA_001491315.1	GCA_005228995.1	GCA_008153965.1	GCA_008334925.1	GCA_008310575.1	GCA_008349205.1
GCA_001492415.1	GCA_005223655.1	GCA_008154165.1	GCA_008350525.1	GCA_008308255.1	GCA_008347925.1
GCA_001493015.1	GCA_005228235.1	GCA_008125365.1	GCA_008011635.1	GCA_008290995.1	GCA_008350225.1
GCA_001496175.1	GCA_000314245.1	GCA_008128025.1	GCA_008229675.1	GCA_007898025.1	GCA_008349665.1
GCA_001499055.1	GCA_000314185.1	GCA_008276685.1	GCA_007895695.1	GCA_007899345.1	GCA_008233885.1
GCA_001496795.1	GCA_005262265.1	GCA_008279205.1	GCA_007904425.1	GCA_007904845.1	GCA_008275445.1
GCA_001419445.1	GCA_005241705.1	GCA_008282795.1	GCA_007900325.1	GCA_007966945.1	GCA_008125865.1
GCA_001487795.1	GCA_001236025.1	GCA_008283035.1	GCA_008134005.1	GCA_007987225.1	GCA_008153405.1
GCA_001491535.1	GCA_005227415.1	GCA_008283995.1	GCA_007940365.1	GCA_008161065.1	GCA_008220505.1
GCA_001492635.1	GCA_005224215.1	GCA_008287355.1	GCA_007941885.1	GCA_008133545.1	GCA_007960385.1
GCA_001493795.1	GCA_005224515.1	GCA_008287035.1	GCA_007945105.1	GCA_008344245.1	GCA_008219065.1
GCA_001494615.1	GCA_005227735.1	GCA_008288375.1	GCA_007946125.1	GCA_007940385.1	GCA_007985445.1
GCA_001495655.1	GCA_005240445.1	GCA_008293775.1	GCA_007967465.1	GCA_007963285.1	GCA_008211105.1
GCA_001499555.1	GCA_005226335.1	GCA_008332705.1	GCA_008209325.1	GCA_007964145.1	GCA_008216745.1
GCA_001496155.1	GCA_005264215.1	GCA_008334485.1	GCA_008236025.1	GCA_008284295.1	GCA_009254285.1
GCA_001498795.1	GCA_005243485.1	GCA_008334905.1	GCA_007897545.1	GCA_008287535.1	GCA_007947425.1
GCA_001419425.1	GCA_005242405.1	GCA_008336445.1	GCA_007899895.1	GCA_007966505.1	GCA_007950325.1
GCA_001419545.1	GCA_005230355.1	GCA_008336585.1	GCA_007920305.1	GCA_007985265.1	GCA_008210525.1
GCA_005145645.1	GCA_005230455.1	GCA_008338465.1	GCA_007984205.1	GCA_008349415.1	GCA_008142445.1
GCA_003590895.1	GCA_005230255.1	GCA_008339005.1	GCA_008228305.1	GCA_007898965.1	GCA_008142905.1
GCA_003590915.1	GCA_005231815.1	GCA_008341505.1	GCA_007931105.1	GCA_007906035.1	GCA_008332585.1
GCA_003590965.1	GCA_005241005.1	GCA_008340225.1	GCA_007945575.1	GCA_007930975.1	GCA_007956625.1

GCA_005145655.1	GCA_005226055.1	GCA_008341725.1	GCA_007966745.1	GCA_007946245.1	GCA_008136005.1
GCA_005145685.1	GCA_005240765.1	GCA_008344585.1	GCA_000146835.1	GCA_007947145.1	GCA_008339225.1
GCA_001704895.1	GCA_005242565.1	GCA_008344925.1	GCA_008232805.1	GCA_007947665.1	GCA_007901005.1
GCA_001545425.1	GCA_005241525.1	GCA_008346365.1	GCA_007940965.1	GCA_007987965.1	GCA_007900125.1
GCA_001545445.1	GCA_005241745.1	GCA_008350375.1	GCA_007932905.1	GCA_008238045.1	GCA_007960425.1
GCA_004368265.1	GCA_005228575.1	GCA_008350025.1	GCA_007940025.1	GCA_008135325.1	GCA_007971745.1
GCA_000254015.2	GCA_005263395.1	GCA_001224645.1	GCA_007941305.1	GCA_008126885.1	GCA_007983415.1
GCA_000253995.2	GCA_005241465.1	GCA_001228685.1	GCA_007957445.1	GCA_008287435.1	GCA_007983345.1
GCA_000254035.2	GCA_005228075.1	GCA_001232625.1	GCA_008134145.1	GCA_008335185.1	GCA_008219765.1
GCA_000254055.2	GCA_005263575.1	GCA_001234005.1	GCA_007932705.1	GCA_007946425.1	GCA_008141305.1
GCA_000254075.2	GCA_005242905.1	GCA_001235265.1	GCA_008228045.1	GCA_008208045.1	GCA_008154805.1
GCA_000254115.2	GCA_005231515.1	GCA_001235605.1	GCA_008291315.1	GCA_008208585.1	GCA_008296215.1
GCA_000254155.2	GCA_005240405.1	GCA_008892025.1	GCA_008227845.1	GCA_008233945.1	GCA_008347485.1
GCA_000254175.2	GCA_005240485.1	GCA_001231465.1	GCA_008126485.1	GCA_008240605.1	GCA_008239365.1
GCA_000254215.2	GCA_005223795.1	GCA_001232305.1	GCA_007983865.1	GCA_008152145.1	GCA_008291595.1
GCA_003996815.1	GCA_006349525.1	GCA_001225085.1	GCA_008127825.1	GCA_008336045.1	GCA_008305595.1
GCA_001545295.1	GCA_006349535.1	GCA_006370305.1	GCA_008222425.1	GCA_007934985.1	GCA_008331645.1
GCA_004329325.1	GCA_005231635.1	GCA_005228335.1	GCA_008227505.1	GCA_007944025.1	GCA_008338385.1
GCA_000253975.2	GCA_005226435.1	GCA_006370255.1	GCA_007893545.1	GCA_007947565.1	GCA_007953785.1
GCA_000253915.2	GCA_007906165.1	GCA_006370345.1	GCA_007900925.1	GCA_008287575.1	GCA_008209405.1
GCA_000494775.1	GCA_007921175.1	GCA_005226295.1	GCA_007930505.1	GCA_008332885.1	GCA_008231885.1
GCA_002118015.1	GCA_007921895.1	GCA_008236365.1	GCA_007935705.1	GCA_008333145.1	GCA_008219625.1
GCA_000253955.3	GCA_007928755.1	GCA_008217505.1	GCA_007943165.1	GCA_008152205.1	GCA_008228185.1
GCA_001765195.1	GCA_007932645.1	GCA_008220185.1	GCA_007964585.1	GCA_008131125.1	GCA_008228385.1
GCA_000254195.2	GCA_007936635.1	GCA_006348485.1	GCA_007964935.1	GCA_007951795.1	GCA_008208135.1
GCA_000254135.2	GCA_007940065.1	GCA_006349405.1	GCA_007970305.1	GCA_008135665.1	GCA_008209085.1
GCA_000954195.1	GCA_007939345.1	GCA_007897345.1	GCA_007985245.1	GCA_007945285.1	GCA_008213725.1
GCA_006346685.1	GCA_007941705.1	GCA_008214645.1	GCA_007981555.1	GCA_007983545.1	GCA_008217285.1
GCA_006346985.1	GCA_007944825.1	GCA_007986225.1	GCA_007984405.1	GCA_007985985.1	GCA_008213925.1
GCA_006347455.1	GCA_007942805.1	GCA_008227425.1	GCA_007988405.1	GCA_007898765.1	GCA_006348005.1
GCA_006346865.1	GCA_007944445.1	GCA_006348115.1	GCA_008219185.1	GCA_006348495.1	GCA_008227665.1
GCA_006347645.1	GCA_007987465.1				

Table S3. *Campylobacter coli* strains from slaughterhouse and retail market samples.

Sample IDs	Strain IDs	Sources	MLST STs	MLST CCs	Aerotolerance Levels*
Sample01	Strain01	Duck slaughter house	829	828	HAT
Sample02	Strain02		829	828	HAT
Sample03	Strain03		829	828	AT
Sample04	Strain04		1593	828	HAT
Sample05	Strain05		1593	828	AT
Sample06	Strain06		855	828	AT
Sample07†	Strain07		829	828	AT
Sample07†	Strain08		855	828	AT
Sample08	Strain09		829	828	AT
Sample09	Strain10		829	828	AT
Sample10†	Strain11		829	828	HAT
Sample10†	Strain12		832	828	HAT
Sample11	Strain13		855	828	AT
Sample12	Strain14		832	828	HAT
Sample13	Strain15		829	828	AT
Sample14	Strain16		6184	828	HAT
Sample15†	Strain17		855	828	HAT
Sample15†	Strain18		855	828	AT
Sample16	Strain19		855	828	AT
Sample17	Strain20		855	828	HAT
Sample18†	Strain21		832	828	HAT
Sample18†	Strain22		832	828	HAT
Sample19†	Strain23		855	828	HAT
Sample19†	Strain24		855	828	AT
Sample20	Strain25		1593	828	AT
Sample21	Strain26		855	828	HAT
Sample22	Strain27		855	828	HAT
Sample23	Strain28	Retail markets	855	828	HAT
Sample24	Strain29		9575	828	OS
Sample25	Strain30		9575	828	OS
Sample26	Strain31		832	828	AT
Sample27	Strain32		902	828	HAT
Sample28	Strain33		5507	828	OS
Sample29	Strain34		830	828	HAT
Sample30	Strain35		828	828	HAT
Sample31	Strain36		2711	828	AT
Sample32	Strain37		9867	828	AT

Sample33	Strain38	830	828	HAT
Sample34	Strain39	1055	828	HAT
Sample35	Strain40	830	828	HAT
Sample36	Strain41	7818	828	OS
Sample37	Strain42	827	828	HAT
Sample38	Strain43	1586	828	AT
Sample39	Strain44	860	828	AT
Sample40	Strain45	830	828	HAT
Sample41[†]	Strain46	6148	UA	HAT
Sample41[†]	Strain47	860	828	AT
Sample42	Strain48	6148	UA	OS
Sample43	Strain49	860	828	OS
Sample44	Strain50	830	828	AT
Sample45	Strain51	860	828	HAT
Sample46	Strain52	829	828	HAT
Sample47	Strain53	829	828	AT
Sample48	Strain54	860	828	AT
Sample49	Strain55	860	828	HAT
Sample50	Strain56	830	828	HAT

* OS, oxygen-sensitive; AT, aerotolerant; HAT, hyper-aerotolerant. † Duck samples harboring epidemiologically-unrelated *C. coli* isolates.

Table S4. Characteristics of epidemiologically-unrelated *Campylobacter coli* strains isolated from the same duck samples

Sample IDs	Strain IDs	MLST STs (CCs)	Virulence gene patterns	AMR patterns	Aerotolerance levels
Sample07	Strain07	829 (828)	*cadF-flaA-cdtA-wlaN-ceuE*	CIP-TET-NAL	AT
	Strain08	855 (828)	*cadF-flaA-cdtA-wlaN-ceuE*	CIP-NAL	AT
Sample10	Strain11	829 (828)	*cadF-flaA-cdtA-wlaN-ceuE*	CIP-NAL	HAT
	Strain12	832 (828)	*cadF-cdtA-wlaN-ceuE*	CIP-TET-STR-NAL	HAT
Sample15	Strain17	855 (828)	*cadF-flaA-cdtA-wlaN-ceuE*	CIP-TET-STR-NAL	HAT
	Strain18	855 (828)	*cadF-flaA-cdtA-wlaN-ceuE*	CIP-TET-NAL	AT
Sample18	Strain21	832 (828)	*flaA*	CIP-TET-NAL	HAT
	Strain22	832 (828)	*cdtA-wlaN-ceuE*	CIP-TET-NAL	HAT
Sample19	Strain23	855 (828)	*cadF-flaA-cdtA-wlaN-ceuE*	CIP-TET-NAL	HAT
	Strain24	855 (828)	*cadF-flaA-cdtA-wlaN-ceuE*	CIP-TET-NAL	AT
Sample41	Strain46	6148 (UA)	*virB11-cadF-flaA-cdtA-wlaN-ceuE*	CIP-TET-NAL	HAT
	Strain47	860 (828)	*cadF-flaA-hcp-cdtA-iamA-wlaN*	CIP-TET-STR-NAL	AT

Table S5. Antimicrobial resistance rates according to aerotolerance levels of *Campylobacter coli* isolates from duck sources

Aerotolerance Levels[*]	*n*	Antimicrobial[†] Resistance Rates (%)								
		ERY	CHL	CIP	TET	TEL	GEN	AZI	STR	NAL
OS	6	33.3%	0%	83.3%	100%	33.3%	33.3%	33.3%	50%	83.3%
AT	22	4.5%	0%	100%	77.3%	4.5%	4.5%	4.5%	13.6%	100%
HAT	28	7.1%	0%	100%	67.9%	10.7%	3.6%	7.1%	28.6%	100%
Total	56	8.9%	0%	98.2%	75%	10.7%	7.1%	8.9%	25%	98.2%

* OS, oxygen-sensitive; AT, aerotolerant; HAT, hyper-aerotolerant. † ERY, erythromycin; CHL, chloramphenicol; CIP, ciprofloxacin; TET, tetracycline; TEL, telithromycin; GEN, gentamicin; AZI, azithromycin; STR, streptomycin; NAL, nalidixic acid.

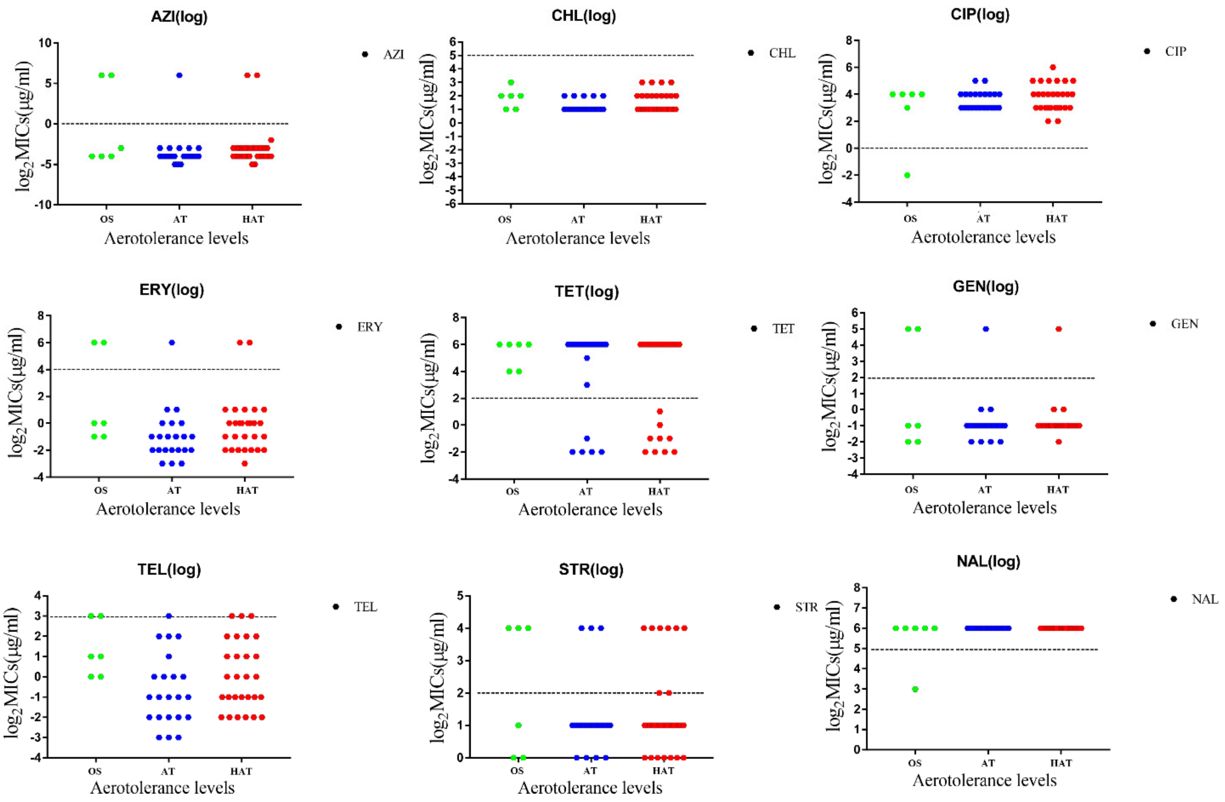

Figure S1. Minimum inhibitory concentration (MIC) values of each antimicrobial against *Campylobacter coli* isolates according to aerotolerance levels. The MIC values of each antimicrobial against *C. coli* isolates from duck sources are expressed as log2MICs (µg/mL). Dotted lines indicate break points, expressed as log value, for resistance to each antimicrobial in *C. coli* isolates. OS, oxygen-sensitive (green); AT, aerotolerant (blue); HAT, hyper-aerotolerant (red). ERY, erythromycin; CHL, chloramphenicol; CIP, ciprofloxacin; TET, tetracycline; TEL, telithromycin; GEN, gentamicin; AZI, azithromycin; STR, streptomycin; NAL, nalidixic acid.

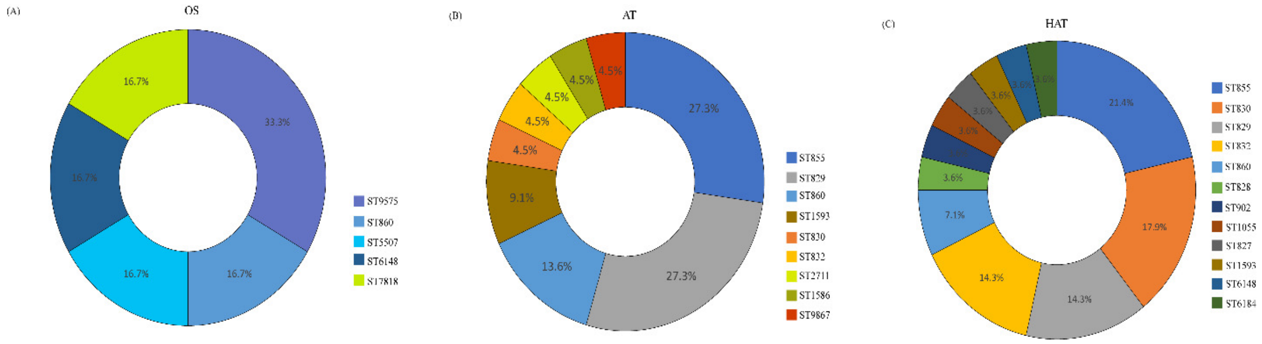

Figure S2. Multi-locus sequence typing (MLST) analysis according to aerotolerance levels of *Campylobacter coli* isolates from duck sources. In total, 18 MLST sequence types (STs) were identified. (**A**) MLST STs of OS *C. coli* isolates. (**B**) MLST STs of AT *C. coli* isolates. (**C**) MLST STs of HAT *C. coli* isolates. Most MLST STs in each group were different (OS: ST9575, AT: ST829 and ST855, HAT: ST855). ST855 was the predominant MLST genotype in our isolates. OS, oxygen-sensitive; AT, aerotolerant; HAT, hyper-aerotolerant.

References

1. Yamazaki-Matsune, W.; Taguchi, M.; Seto, K.; Kawahara, R.; Kawatsu, K.; Kumeda, Y.; Kitazato, M.; Nukina, M.; Misawa, N.; Tsukamoto, T. Development of a multiplex PCR assay for identification of Campylobacter coli, Campylobacter fetus, Campylobacter hyointestinalis subsp. hyointestinalis, Campylobacter jejuni, Campylobacter lari and Campylobacter upsaliensis. *Journal of Medical Microbiology* **2007**, *56*, 1467-1473.
2. Nachamkin, I.; Ung, H.; Patton, C.M. Analysis of HL and O serotypes of Campylobacter strains by the flagellin gene typing system. *Journal of clinical microbiology* **1996**, *34*, 277-281.
3. Koolman, L.; Whyte, P.; Burgess, C.; Bolton, D. Distribution of virulence-associated genes in a selection of Campylobacter isolates. *Foodborne pathogens and disease* **2015**, *12*, 424-432.
4. Konkel, M.E.; Gray, S.A.; Kim, B.J.; Garvis, S.G.; Yoon, J. Identification of the EnteropathogensCampylobacter jejuni and Campylobacter coli Based on the cadF Virulence Gene and Its Product. *Journal of clinical microbiology* **1999**, *37*, 510-517.
5. Datta, S.; Niwa, H.; Itoh, K. Prevalence of 11 pathogenic genes of Campylobacter jejuni by PCR in strains isolated from humans, poultry meat and broiler and bovine faeces. *J Med Microbiol* **2003**, *52*, 345-348, doi:10.1099/jmm.0.05056-0.
6. Müller, J.; Schulze, F.; Müller, W.; Hänel, I. PCR detection of virulence-associated genes in Campylobacter jejuni strains with differential ability to invade Caco-2 cells and to colonize the chick gut. *Veterinary microbiology* **2006**, *113*, 123-129.
7. Gonzalez, I.; Grant, K.A.; Richardson, P.T.; Park, S.F.; Collins, M.D. Specific identification of the enteropathogens Campylobacter jejuni and Campylobacter coli by using a PCR test based on the ceuE gene encoding a putative virulence determinant. *Journal of clinical microbiology* **1997**, *35*, 759-763.
8. Bang, D.D.; Scheutz, F.; Ahrens, P.; Pedersen, K.; Blom, J.; Madsen, M. Prevalence of cytolethal distending toxin (cdt) genes and CDT production in Campylobacter spp. isolated from Danish broilers. *Journal of medical microbiology* **2001**, *50*, 1087-1094.
9. Bang, D.D.; Nielsen, E.M.; Scheutz, F.; Pedersen, K.; Handberg, K.; Madsen, M. PCR detection of seven virulence and toxin genes of Campylobacter jejuni and Campylobacter coli isolates from Danish pigs and cattle and cytolethal distending toxin production of the isolates. *Journal of applied microbiology* **2003**, *94*, 1003-1014.

10. Bleumink-Pluym, N.M.; van Alphen, L.B.; Bouwman, L.I.; Wösten, M.M.; van Putten, J.P. Identification of a functional type VI secretion system in Campylobacter jejuni conferring capsule polysaccharide sensitive cytotoxicity. *PLoS pathogens* **2013**, *9*, e1003393.
11. Corcionivoschi, N.; Gundogdu, O.; Moran, L.; Kelly, C.; Scates, P.; Stef, L.; Cean, A.; Wren, B.; Dorrell, N.; Madden, R.H. Virulence characteristics of hcp+ Campylobacter jejuni and Campylobacter coli isolates from retail chicken. *Gut pathogens* **2015**, *7*, 20.
12. Bacon, D.J.; Alm, R.A.; Burr, D.H.; Hu, L.; Kopecko, D.J.; Ewing, C.P.; Guerry, P. Involvement of a plasmid in virulence of Campylobacter jejuni 81-176. *Infection and immunity* **2000**, *68*, 4384-4390.
